# Robot-Assisted Retroperitoneal Tumor Resection: Evaluation of Its Safety, Feasibility, and Implementation in a Japanese Clinical Setting

**DOI:** 10.7759/cureus.110801

**Published:** 2026-06-13

**Authors:** Yoshiyuki Matsui

**Affiliations:** 1 Department of Urology, National Cancer Center Hospital, Tokyo, JPN

**Keywords:** feasibility and safety, retroperitoneal tumor, robotic surgery, surgical implementation, tumor size

## Abstract

Background

Robot-assisted resection of retroperitoneal tumors has been increasingly reported worldwide; however, its clinical implementation in Japan remains limited and is not yet approved under the national health insurance system. We aimed to evaluate the safety and feasibility of this approach in a Japanese clinical setting.

Methods

We retrospectively reviewed seven consecutive patients who underwent robot-assisted retroperitoneal tumor resection at a high-volume cancer center between July 2024 and September 2025. Patient selection was based on tumor size, location, and absence of extensive vascular invasion. All procedures were performed by an experienced robotic surgeon. Perioperative outcomes, including operative time, blood loss, conversion rate, complications, and pathological results, were analyzed.

Results

Median tumor diameter was 35 mm (range, 15-81 mm), including three tumors ≥60 mm. All procedures were completed robotically without conversion. No intraoperative complications or transfusions occurred. All patients achieved R0 resection without tumor rupture. Median hospital stay was six days, with no major complications or readmissions. At the 17-month median follow-up, all patients were alive without recurrence.

Conclusions

These findings suggest that robot-assisted resection is a safe and feasible minimally invasive approach in selected patients and support further prospective evaluation and broader implementation in Japan.

## Introduction

Retroperitoneal tumors are rare and heterogeneous lesions arising from mesenchymal or neurogenic tissues in a deep and anatomically complex space. Surgical resection remains the only potentially curative treatment, because complete macroscopic resection is essential for oncological control [1,2]. Open surgery has traditionally been the standard approach because it provides wide exposure and facilitates en bloc resection, especially in cases involving major vessels. However, it is associated with substantial morbidity, including increased blood loss, postoperative pain, and prolonged recovery [3,4].

Minimally invasive approaches have been increasingly adopted to reduce surgical invasiveness. In Japan, conventional laparoscopic surgery is already covered by the national health insurance system and widely used for selected retroperitoneal tumors [5]. However, laparoscopic surgery has technical limitations, including restricted instrument articulation and limited visualization, particularly in the confined retroperitoneal space [6,7], which may limit its applicability to anatomically complex tumors.

Robotic-assisted surgery has been introduced to overcome these limitations, offering enhanced three-dimensional (3D) visualization and improved instrument dexterity. Evidence from other surgical fields suggests that robotic approaches may provide improved perioperative outcomes, including reduced blood loss and complication rates, compared with conventional laparoscopy [8,9]. These advantages may be particularly relevant in retroperitoneal tumor surgery, where meticulous dissection is required to avoid vascular injury and tumor rupture.

Although robotic retroperitoneal tumor resection has been increasingly reported internationally, its implementation in Japan remains limited and is not yet approved under the national health insurance system. In the Japanese healthcare system, the introduction of novel surgical technologies generally requires the accumulation of domestic clinical evidence, even when international data are available. This requirement is particularly critical for rare diseases such as retroperitoneal tumors, where large-scale prospective trials are often impractical.

In parallel, there is a growing demand for de-escalation of surgical invasiveness in oncological care. The development of minimally invasive yet oncologically sound approaches is therefore an important clinical priority. In this context, robot-assisted surgery may represent a promising option to achieve both surgical precision and reduced invasiveness.

Therefore, this study aimed to evaluate the safety and feasibility of robot-assisted retroperitoneal tumor resection in a Japanese clinical setting, with particular emphasis on generating real-world evidence to support its future implementation within the national healthcare system.

## Materials and methods

This retrospective case series included seven consecutive patients who underwent robot-assisted retroperitoneal tumor resection at a high-volume cancer center, the National Cancer Center Hospital in Tokyo, Japan, between July 2024 and September 2025. The study was approved by the Ethics Board of the National Cancer Center Hospital (approval number: 2017-168) and conducted in accordance with institutional ethical standards. Written informed consent for surgery and use of clinical data was obtained from all patients. Among these patients, two underwent surgery as self-funded procedures, whereas five were treated within the national health insurance system following preoperative diagnostic evaluation and institutional approval.

Clinical data, including patient characteristics, tumor features, imaging findings, and perioperative outcomes, were collected from medical records. Preoperative evaluation was performed using contrast-enhanced computed tomography (CT) and/or magnetic resonance imaging (MRI) to assess tumor size, anatomical relationships, and resectability. Patients were selected for robotic surgery based on tumor characteristics, including size and location. Histological diagnosis (benign or malignant) was not used as a selection criterion. Giant tumors were excluded to ensure adequate working space for robotic manipulation within the abdominal cavity. Although no predefined size cutoff was applied, tumors larger than approximately 10 cm in diameter were generally considered unsuitable for a robotic approach on our institutional experience, technical judgment, and previous literature [5]. In addition, cases with apparent major vascular invasion requiring vascular resection and reconstruction or those requiring combined intestinal resection with intestinal reconstruction were considered unsuitable because of the increased technical complexity for complete tumor resection. Robot-assisted surgery was therefore offered to patients with retroperitoneal tumors without these features after obtaining informed consent.

All procedures were performed by an experienced robotic surgeon using either a multi-port or single-port robotic platform via a transperitoneal approach. Under general anesthesia, patient positioning and port placement were tailored according to tumor location. The surgical procedure consisted of careful dissection under magnified three-dimensional visualization, with particular attention to preserving adjacent organs and major vascular structures. En bloc resection was attempted in all cases while avoiding tumor rupture.

Perioperative outcomes included operative time, estimated blood loss, conversion to open surgery, postoperative hospital course including inflammatory index (C-reactive protein (CRP)), postoperative hospital stay, and complications (graded according to the Clavien-Dindo classification) and pathological outcomes to assess the surgical safety and oncological adequacy. 　

Descriptive statistics were used to summarize the patients’ characteristics, with continuous variables presented as medians (interquartile range (IQR)) and categorical variables presented as frequencies and proportions. The Student's t test was used to compare normally distributed variables between groups, whereas the Mann-Whitney U test was used for non-normally distributed variables. Categorical data were compared using the chi-squared test. All analyses were performed using EZR (Saitama Medical Center, Jichi Medical University, Saitama, Japan) [10], with a two-sided significance threshold of p = 0.05.

## Results

**Figure 1:**
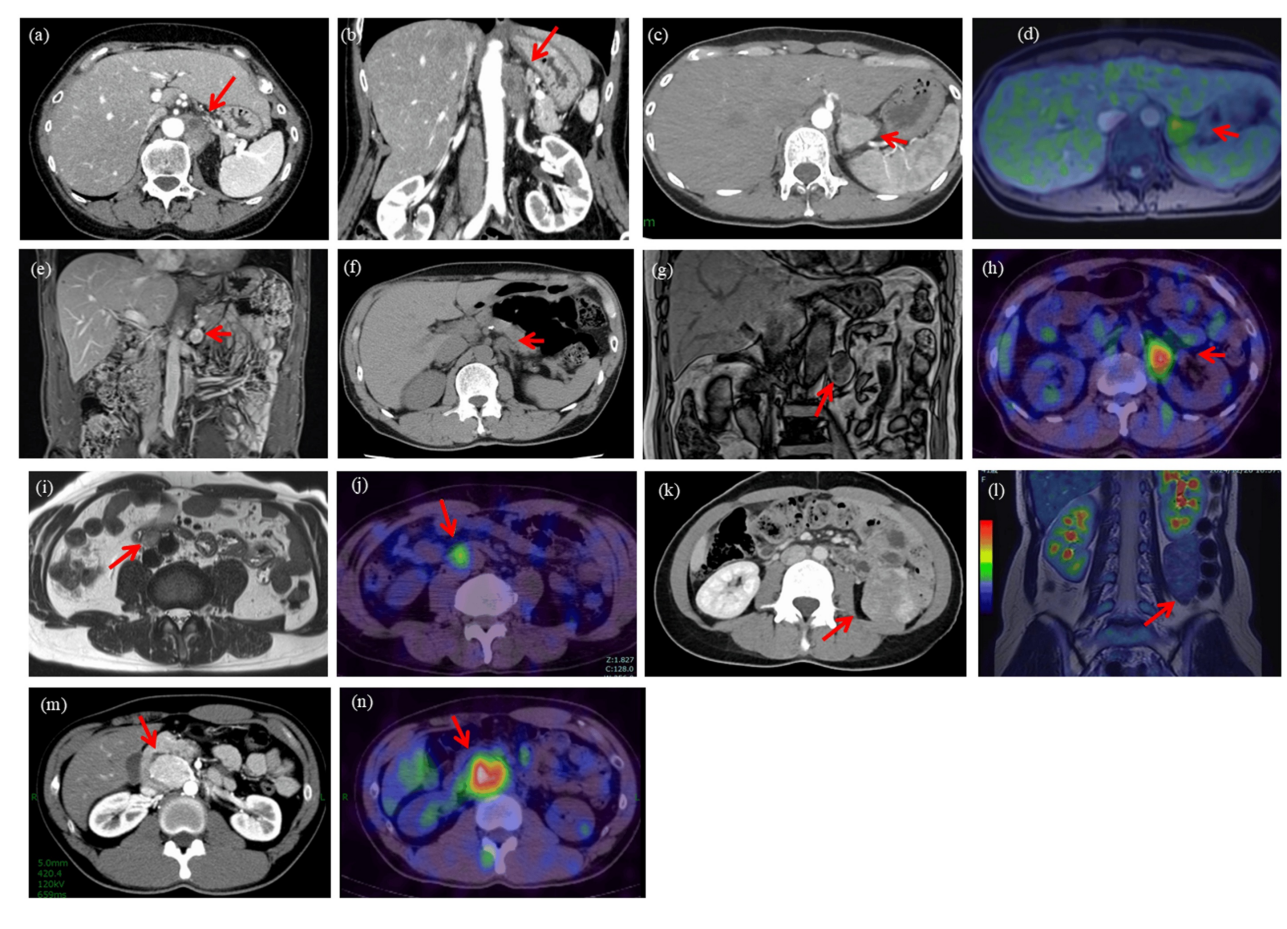
Preoperative imaging findings of all tumors (red arrows) (a, b) Case 1: preoperative axial and coronal CT images showing a tumor surrounding the left renal vessels on the left side of the aorta; (c, d) Case 2: preoperative axial CT and FDG-PET images; (e, f) Case 3: preoperative coronal MRI and axial CT images; (g, h) Case 4: preoperative coronal MRI and axial I123-MIBG scintigraphy images; (i, j) Case 5: preoperative axial MRI and I123-MIBG scintigraphy images; (k, l) Case 6: preoperative axial CT and coronal FDG-PET images; (m, n) Case 7: preoperative axial CT and I123-MIBG scintigraphy images I123-MIBG: iodine-123 metaiodobenzylguanidine

The cohort consisted of four men and three women, with a median age of 61.7 years (range, 21.6-72.2) (Table 1). All patients had an Eastern Cooperative Oncology Group performance status of 0. The median BMI was 21.6 kg/m² (range, 19.0-23.3). Preoperative clinical diagnoses included adrenocortical carcinoma (n=2), pheochromocytoma (n=2), paraganglioma (n=2), and renal angiomyolipoma (n=1). Tumors were located in anatomically complex retroperitoneal regions, including the renal hilum, para-adrenal space, para-aortic region, interaortocaval space, and peripancreatic area. The median tumor diameter was 35 mm (range, 15-81 mm), including three tumors measuring ≥60 mm (Figure 1).

**Table 1:** Case summaries BMI: body mass index; CRP: C-reactive protein; POD: postoperative day; F: female; M: male; MP: multi-ports; SP: single port

Case	Age (years)	Sex	BMI (kg/m^2^)	Clinical diagnosis	Tumor size (mm)	Tumor location	Previous abdominal surgery	Robot type	Operation time (minutes)	Console time (minutes)	Blood loss (ml)	Complication	Pathological diagnosis	CRP POD1 (mg/dl)	CRP POD3 (mg/dl)	Time to regular diet (days)	Postoperative length of stay (days)	Follow-up periods (month)
1	62	F	19.4	Adrenocortical carcinoma	81	Left renal vessels	None	MP	216	181	8	-	Diffuse large B-cell lymphoma	4.03	6.94	2	5	20.4
2	61	F	19.0	Metastatic adrenal tumor	30	Cranial to left renal vessels	Appendectomy	MP	65	45	4	-	Castleman disease	0.34	0.71	2	6	19.6
3	64	M	21.6	Pheochromocytoma	15	Para-aorta	None	MP	135	115	8	-	Paraganglioma	0.44	1.86	2	5	18.2
4	72	M	23.3	Pheochromocytoma	35	Dorsal to pancreas	None	MP	134	110	40	Right subclavian vein thrombosis	Paraganglioma	2.88	9.40	1	7	17.0
5	66	M	22.2	Paraganglioma	20	Inter-aortocaval	None	MP	61	35	0	-	Paraganglioma	3.83	2.06	2	4	15.2
6	42	F	20.1	Renal angiomyolipoma	60	Caudal to left kidney	None	SP	127	111	0	-	Leiomyoma	0.35	0.48	3	7	13.3
7	22	M	21.6	Paraganglioma	60	Inter-aortocaval	none	MP	151	110	29	-	Paraganglioma	2.07	1.21	2	8	6.7

Robot-assisted retroperitoneal tumor resection was successfully completed in all seven patients. In the six multi-port cases, surgery was performed in the lateral decubitus position. Tumors located paracaval or interaortocaval were approached using the same port configuration as that used for right partial nephrectomy using the Kocher maneuver, which involves mobilizing the hepatic flexure and ascending colon, whereas tumors located in the para-aortic region were managed using the same port configuration as that used for left partial nephrectomy, mobilizing the splenic flexure of the colon and dividing the gastrocolic ligament and splenorenal ligament [11]. 

Using these procedures, the retroperitoneal structures, including the posterior portion of the pancreatic head, tail of the pancreas, bilateral adrenal glands, bilateral renal veins, inferior vena cava (IVC), and the aorta, could be identified. Dissection and mobilization from the adjacent structures were performed using a robotic fenestrated bipolar coagulation, monopolar scissors, and ProGrasp™ Forceps. The feeding vessels were isolated and clipped with OMNIFinger™ clips (Grena Ltd, Nottingham, United Kingdom), or by LigaSure™ (Medtronic plc, Galway, Ireland) sutures and divided (In the single-port case, surgery was performed in the lateral decubitus position. A 4-cm skin incision was made 2 cm from the ipsilateral anterior superior iliac spine for placement of the access port, and one additional 12-mm assistant port was used separately.

Despite the anatomical complexity and tumor size, no intraoperative complications occurred, and no procedures required conversion to open or conventional laparoscopic surgery. Combined organ resection was not required in any case, except for one patient who underwent partial resection of the descending colon wall with a surgical stapler (case 6). The median operative time was 134 minutes (IQR, 96-143), the median console time was 110 minutes (IQR, 78-113), and the median estimated blood loss was 8 mL (IQR, 2-19) (Table 2). Estimated blood loss was minimal in all patients, and no perioperative blood transfusions were required. Even in those with paraganglioma, intraoperative blood pressure fluctuations remained within an acceptable range, and no major hemodynamic complications occurred with the use of antihypertensive and vasopressor agents before and after tumor resection. All patients achieved R0 resection, and no intraoperative or pathological tumor rupture was observed. Although estimated blood loss was similar between patients with tumors <60 mm and those with tumors ≥60 mm, larger tumors tended to require longer operative times. Given the small sample size, no definitive conclusions can be drawn; nevertheless, tumor size may represent an important factor when considering the indication for robot-assisted retroperitoneal tumor resection.

**Table 2:** Demographic intraoperative and perioperative data of all cases. CRP: C-reactive protein; POD: postoperative day; LOS: length of stay

Item	Median	Q1 (1st Quartile)	Q3 (3rd Quartile)	IQR (Interquartile Range)
Operation Time (min)	134	96	143	47
Console Time (min)	110	77.5	113	35.5
Blood Loss (ml)	8	2	18.5	16.5
CRP POD 1 (mg/dL)	2.07	0.395	3.355	2.96
CRP POD 3 (mg/dL)	1.86	0.96	4.5	3.54
Time to Regular Diet (days)	2	2	2	0
Postoperative LOS (days)	6	5	7	2
Tumor Size (mm)	35	25	60	35

Postoperative recovery was uneventful in all patients (Tables 1, 2). Oral intake was resumed at a median of POD 2 (range, 1-3), and the median duration of fasting was two days. The median postoperative hospital stay was six days (range, 4-8). Postoperative inflammatory response was mild. Median CRP levels were 2.07 mg/dL (IQR, 0.4-3.36) on POD 1 and 2.06 mg/dL (IQR, 0.96-4.5) on POD 3. One patient developed right subclavian vein thrombosis requiring treatment with direct oral anticoagulants, which was classified as a Clavien-Dindo grade II complication. But no patients experienced severe postoperative complications (Clavien-Dindo grade ≥III), and no readmissions occurred within 30 days.

**Figure 2:**
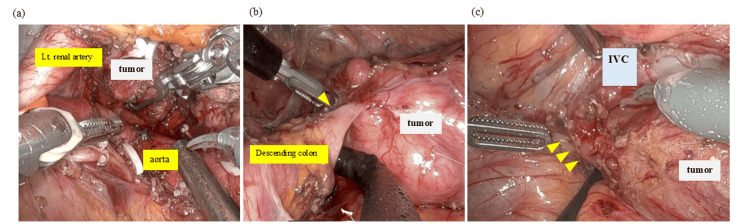
Representative intraoperative robotic view of case 1 (a), case 6 (b), and case 7 (c) A clear operative field of (a) case 1 was maintained even within the narrow space between the aorta and the tumor; (b) case 6 shows the adhesion between the tumor and descending colon; (c) case 7 shows the main drainage vein (yellow arrowheads) from the tumor (paraganglioma) to the inferior vena cava (IVC) during tumor dissection.

Final pathological diagnoses were paraganglioma (n=4), Diffuse large B-cell lymphoma (n=1), Castleman disease (n=1), and retroperitoneal leiomyoma (n=1). Complete tumor excision with negative margins was achieved in all patients. At a median follow-up of 17 months (range, 7-20), all patients were alive without evidence of local recurrence or distant metastasis. Representative surgical figures of cases 1, 6, and 7, and short summary surgical videos of cases 1 and 7 are shown in Figure 2 and Videos 1, 2.

**Video 1:** Short summary video of Case 1

**Video 2:** Short summary video of Case 7

## Discussion

In this initial experience, robot-assisted retroperitoneal tumor resection was safely and feasibly performed without conversion to open surgery or major complications. In particular, tumors adjacent to major vessels were successfully managed using meticulous dissection along the vascular adventitia and early control of feeding vessels in our case series. Although the multi-port approach was used in most cases because of its flexibility for instrument triangulation and vascular control, the single-port approach was also successfully applied in one selected patient with a relatively small and localized tumor. Furthermore, as institutional experience accumulated, a stepwise operative strategy was gradually standardized, including patient positioning, laterality-based port placement, vascular identification, tumor mobilization, and specimen retrieval. While the present study was limited by its small sample size, this standardized approach may have contributed to the favorable perioperative outcomes observed in our series.

These results are consistent with previous reports demonstrating the safety of robotic approaches for retroperitoneal tumors [12-14]. The retroperitoneal space presents unique technical challenges due to its narrow working space and proximity to major vascular structures. Theoretically, robotic systems may facilitate enhanced visualization and instrument articulation, enabling precise dissection and improved control in such anatomically complex regions [15].

Compared with conventional minimally invasive laparoscopic surgery, previous reports have suggested that robotic surgery may offer advantages in terms of reduced blood loss, lower complication rates, and improved surgical precision, as demonstrated in adrenal and colorectal surgery [8,9]. These advantages may be particularly relevant in retroperitoneal tumor surgery, where meticulous dissection is required to avoid vascular injury and tumor rupture.

Accumulating international evidence initially established the feasibility and safety of robotic surgery primarily for benign retroperitoneal tumors. An early consecutive case series of 25 benign nonadrenal retroperitoneal tumors demonstrated that all procedures were completed robotically without conversion or blood transfusion, despite including patients with tumors larger than 5 cm and tumors adherent to major vessels [16]. The authors further reported that neither larger tumor size nor vascular adjacency significantly worsened perioperative outcomes, supporting the technical advantages of robotic surgery in the confined retroperitoneal space. Subsequently, a propensity score-matched study comparing robotic and open surgery for benign retroperitoneal tumors showed that robotic surgery significantly reduced blood loss and postoperative hospital stay without increasing perioperative morbidity or transfusion rates. Importantly, these benefits were maintained even in patients with tumors adjacent to major vessels or with relatively large tumors [17].

More recently, the indications for robotic surgery have expanded to include selected malignant retroperitoneal tumors. A large single-center series of 105 robotic resections reported that 82.9% of procedures were completed without conversion to open surgery [12]. However, larger tumor size, particularly exceeding approximately 6-7 cm, and malignant pathology were identified as independent risk factors for open conversion. In addition, propensity score-matched analyses including tumors involving major vessels demonstrated lower blood loss and shorter postoperative hospital stay with robotic surgery compared with open surgery, even among selected malignant cases [18]. These findings suggest that robotic resection may be feasible even for complex or malignant retroperitoneal tumors in experienced centers, although careful patient selection remains essential because tumor size and malignant features are associated with an increased likelihood of conversion to open surgery. Although no conversion to open surgery was required in our series, operative time tended to increase in tumors larger than 6 cm, suggesting that tumor size is an important factor when considering indications for robotic surgery. Furthermore, for future application of robotic surgery to malignant retroperitoneal tumors such as sarcomas, additional investigation regarding oncological safety, including the potential risk of tumor dissemination associated with robotic manipulation, will be essential.

Despite these encouraging international data, the implementation of robotic retroperitoneal tumor resection in Japan remains limited. One major reason is the structure of the national health insurance system, which requires domestic clinical evidence to support approval and reimbursement of new technologies. In this context, the present study provides important real-world data derived from a Japanese high-volume cancer center. Because of the rarity and heterogeneity of retroperitoneal tumors, conducting large-scale randomized controlled trials in this field is challenging in Japan, and clinical evidence often relies on case series and retrospective analyses. Similar pathways have been observed in the adoption of other surgical innovations in rare conditions, where the accumulation of institutional experiences has played a pivotal role in establishing clinical feasibility prior to broader acceptance.

From a clinical perspective, there is an increasing emphasis on de-escalation of treatment-related invasiveness in oncology. Robot-assisted surgery may contribute to this paradigm shift by enabling precise tumor resection while minimizing surgical trauma. The favorable perioperative outcomes observed in this study, including minimal blood loss, absence of major complications, and short hospital stay, support this concept. Because this was our initial experience with robotic retroperitoneal tumor surgery, we excluded highly complex cases requiring combined vascular resection or reconstruction. Nevertheless, the present study provides preliminary support for the potential utility of the robotic platform in selected retroperitoneal tumors. In particular, the magnified 3D view and enhanced instrument dexterity may have contributed to precise surgical manipulation around major vessels. Whether robotic surgery can be safely extended to more complex retroperitoneal tumors warrants further investigation. However, accumulating international evidence, together with the increasing standardization of robot-assisted surgery for renal cell carcinoma with IVC tumor thrombus in Japan [19], suggests that robotic approaches may be considered for selected complex retroperitoneal tumors at experienced centers.

This study has several limitations. First, the sample size was small, and the retrospective design limits the generalizability of the findings. Second, the follow-up period was relatively short, precluding definitive conclusions regarding long-term oncological outcomes. Third, no direct comparison with open or laparoscopic surgery was performed. Nevertheless, given the regulatory and clinical context in Japan, even small-scale studies such as this play an important role in bridging the gap between technological innovation and clinical implementation.

## Conclusions

Robot-assisted retroperitoneal tumor resection is a feasible and safe minimally invasive approach in selected patients when performed by experienced surgeons, offering significant advantages in blood loss reduction, shorter hospitalization, and surgical precision. Because this study represents our initial experience with robotic retroperitoneal tumor surgery, highly complex cases requiring combined vascular resection or reconstruction were excluded. Further studies with larger cohorts are needed to evaluate surgical feasibility according to tumor biology and anatomical characteristics, including histology, tumor size, tumor location, proximity to major vessels, and the need for adjacent organ resection.
